# Man-Eating Tigers

**Published:** 1860-07

**Authors:** 


*Man-Eating Tigers.*—Since January, 1859, 1,500 Chinese have been carried off by tigers in Johere, the end of the Malacca peninsula. It is now difficult to induce Coolies to work in Johore.

